# Tramadol-Induced Fatal Angioedema: A Rare Case

**DOI:** 10.7759/cureus.64341

**Published:** 2024-07-11

**Authors:** Bhawna Saini, Arohi Agarwal, Gagan Singh, Sreejith Jayachandran, Samyak Jain

**Affiliations:** 1 Clinical Pharmacology and Therapeutics, All India Institute of Medical Sciences, Rishikesh, Rishikesh, IND; 2 Pharmacy Practice, Teerthanker Mahaveer College of Pharmacy, Moradabad, IND; 3 Emergency Medicine, All India Institute of Medical Sciences, Rishikesh, Rishikesh, IND

**Keywords:** fatal angioedema, causality assessment, paracetamol, tramadol, systemic lupus erythematosus

## Abstract

Angioedema is a non-pitting edema that involves the subcutaneous and submucosal layers of the face, lips, neck, oral cavity, larynx, and gut. It may become life-threatening when it involves tissues of the larynx. Angioedema can be triggered by exposure to drugs such as angiotensin-converting enzyme inhibitors (ACE inhibitors), opioid drugs, and nonsteroidal anti-inflammatory drugs (NSAIDs). Tramadol is an opioid analgesic medication that may also induce angioedema, but the incidence of tramadol-induced angioedema is very rare in literature to date. It has been postulated that tramadol may cause fatal angioedema in the presence of underlying diseases such as systemic lupus erythematosus (SLE) or concomitant drugs such as NSAIDs. We describe the case of a patient with SLE who experienced fatal angioedema following tramadol intake.

## Introduction

Angioedema is a non-pitting swelling of the submucosal and subcutaneous tissues that mainly targets the tongue, lips, larynx, face, genitals, and body extremities. It may become life-threatening when it involves the larynx. Angioedema has been broadly classified into hereditary angioedema (HAE) and acquired angioedema (AAE). HAE is caused by the deficiency of the C1 esterase inhibitor (C1 INH) that results in angioedema episodes with variable age of onset of symptoms, usually without exogenous triggers. AAE is usually associated with a lack of family history of angioedema and the presence of anti-C1 INH antibodies associated with lymphoproliferative disorders [1]. AAE can be triggered by drugs such as angiotensin-converting enzyme inhibitors (ACE), opioid drugs, and nonsteroidal anti-inflammatory drugs (NSAIDs). Tramadol is an opioid analgesic medication that has been reported to cause angioedema as an adverse event, although the literature on tramadol-induced angioedema is very sparse. The incidence of tramadol-induced angioedema is reported to vary between 1 in 1,000 and 1 in 10,000 [2]. Tramadol may present with angioedema very rarely, but risk may be enhanced with underlying diseases such as systemic lupus erythematosus (SLE) or concomitant drugs such as NSAIDs [3].

## Case presentation

**Figure 1:**
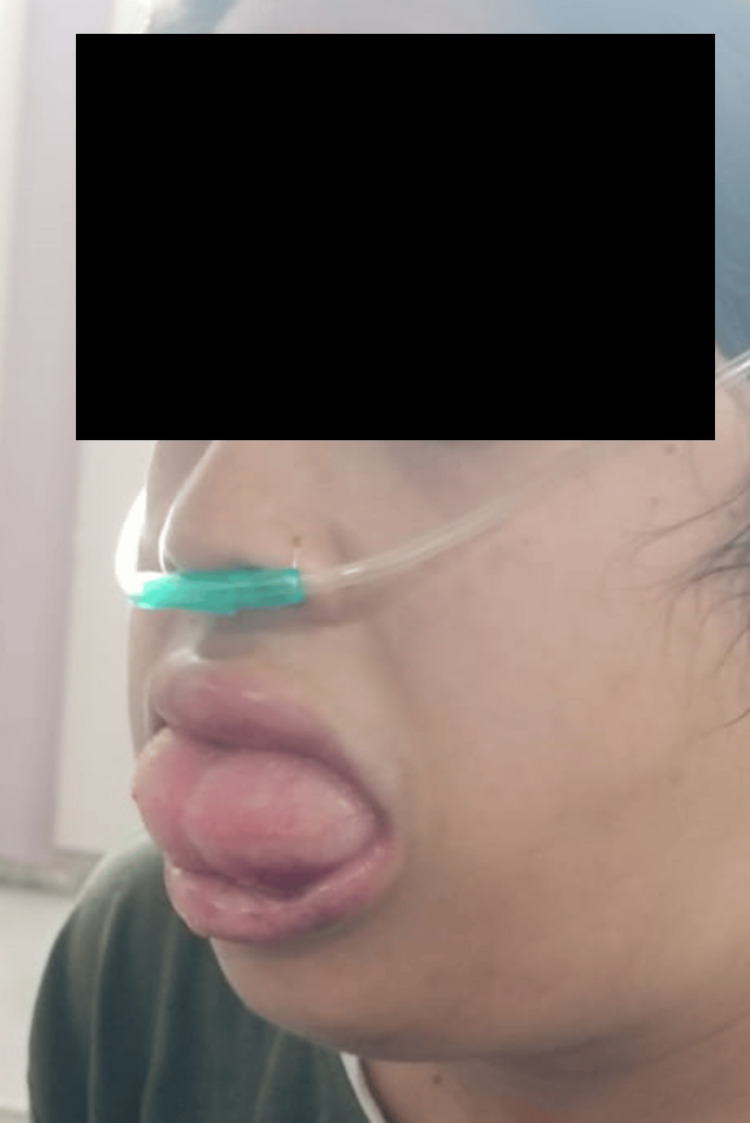
Image of the patient with angioedema

A 23-year-old female patient developed severe shoulder pain without any injury at 3 pm on day 1. She took a fixed dose combination (FDC) of diclofenac 50 mg and paracetamol 325 mg tablet to relieve the pain. The pain was resolved by a single tablet but reappeared after 4 hours. The patient took the same medication again but the shoulder pain did not subside. She was advised an injection of tramadol 50 mg IV at 11 pm in a local hospital to resolve severe shoulder pain refractory to NSAIDs. When the pain did not resolve by the single-dose administration, injection of tramadol 50 mg was re-administered within 30 minutes. When the shoulder pain started resolving, the patient was discharged at 12.10 am at night. The patient started complaining of tongue swelling, but the clinician could not appreciate tongue swelling on examination at discharge. The patient returned home and consumed FDC of paracetamol (325 mg) and tramadol (37.5 mg) tablet after taking a meal at 2.30 am. The patient developed gradually increasing tongue swelling, swollen lips, and rashes with swelling over the face and oral cavity and was admitted to our hospital at 9.30 am on day 2 (Figure 1).

On general examination, the patient was conscious and oriented, with a pulse rate of 70 beats/min, respiratory rate of 20 breaths/min, SPO_2_ of 98% on room air, blood pressure of 128/98 mm Hg, and random blood sugar of 137 mg/dL. The patient's medical history revealed a recent hospital admission 15 days ago with acute febrile illness, pneumonia, pulmonary edema, polyserositis with esophageal candidiasis, and erythematous gastritis with extensive oral ulcers. Her clinical features and previous laboratory reports were suggestive of systematic lupus erythematosus (anti-ds DNA positive, ANA-Hep-2 positive, polyserositis, and pancytopenia) according to the 2019 EULAR/ACR Classification Criteria for Systemic Lupus Erythematosus [4]. The patient had no documented history of drug allergy. The patient was treated immediately with intravenous injection of hydrocortisone 100 mg and 1 ampoule of pheniramine maleate (22.75 mg), as well as nebulization with adrenaline. The patient was recommended for emergency tracheostomy, but her family opted for medical management only. As a result, she received supportive medical treatment. Unfortunately, despite receiving all life-saving measures, the patient passed away the same day due to airway obstruction.

Injection of tramadol 50 mg is a suspected medicine in this case that resulted in angioedema, and the high cumulative dose consumption of tramadol with paracetamol led to fatal angioedema in a female SLE patient. Causality assessment of adverse reaction can be done using the Naranjo scale and the WHO-UMC causality scale.

The Naranjo scale is a scoring method to assess the causality of adverse reaction with exposure to a suspected drug using a simple questionnaire to assign probability scores. Total scores range from -4 to +13; the reaction may be categorized as definite (score ≥ 9), probable (5 to 8), possible (1 to 4), and doubtful (≤ 0). Causality assessment of adverse drug reaction in this patient was possible as per the Naranjo scale (score = 4). This conclusion was based on the temporal occurrence of the reaction following drug administration, which adhered to a recognized pattern for the suspected drug, as well as the presence of a disease that could potentially explain the adverse reaction [5].

Causality assessment of the adverse drug reaction was possible as per WHO-UMC causality scales because of a clear temporal association between adverse reaction and tramadol, pharmacological explanation by published literature evidence, presence of alternate explanation for adverse reaction (SLE as an underlying disease), and absence of information regarding dechallenge and rechallenge of drug in this patient [6].

Hartwig’s severity assessment scale is useful in assessing the severity of adverse drug reaction using seven categories, ranging from level 1 to level 7. Level 1 defines the occurrence of adverse drug reaction without the need of change in treatment with the suspected drug. Level 7 defines the adverse reaction that results into death of the patient. The severity of adverse drug reaction was severe (level 7) as per Hartwig’s severity assessment scale [7].

## Discussion

Tramadol is a centrally acting analgesic that is usually indicated for the treatment of moderate-to-severe pain. It is an opioid analgesic medication that has a dual mechanism of action that includes agonistic effects on the mu-opioid receptor and inhibition of neurotransmitter (serotonin and norepinephrine) reuptake. Tramadol is considered a well-tolerated and safe analgesic that causes fewer adverse effects such as central nervous system and respiratory depression, tachycardia, hypotension, and coma.

In our case report, a female patient with underlying disease SLE gradually developed tongue swelling after injection of tramadol, but this swelling became more prominent after consumption of an FDC of paracetamol (325mg) and tramadol (37.5mg). Hallberg and Brenning reported a similar incidence with 11 cases of angioedema that were possibly related to tramadol with a likely type-I IgE-antibody-mediated mechanism, in which six were serious cases [8]. Keerthana et al. demonstrated a similar event of tramadol-induced angioedema in a female with a history of SLE [3].

The mechanism of development of tramadol-induced angioedema is still not clear, but few studies can generate a picture of the possible mechanism of tramadol-induced angioedema. Tramadol may cause angioedema by its enhanced vasodilating effect. Kaya et al. demonstrated that tramadol induces vasodilatation through the activation of the nitric oxide synthase-guanylate cyclase pathway using isolated rabbit thoracic aortic rings. Tramadol may aggravate vasodilation by increasing nitric oxide production by inhibiting serotonin reuptake, which increases serotonin levels [9].

Our patient also had SLE as an underlying disease. SLE is characterized by multi-organ involvement, autoantibody formations, and the dysregulation of the complement system. SLE manifestations are associated with multiple autoantibodies, ensuing immune complex formation and deposition, and other immune processes. Patients with SLE are likely to experience hypersensitivity reactions including angioedema.

## Conclusions

In this case report, a female patient with SLE disease developed fatal angioedema after tramadol exposure. Tramadol is a suspected drug in this case, with possible association with fatal angioedema episode. Although we have little literature available to support the evidence of tramadol-induced angioedema in an SLE patient, but rapid recognition of such evidence may save a patient’s life with a similar disease. For this purpose, periodic in-hospital training of clinicians will be beneficial. Such patients should be referred to an allergist/immunology specialist for the confirmation of the suspected triggers, prevention advice, and allergen immunotherapy to avoid similar adverse events.
